# Efficacy of *Helicobacter* *pylori* Eradication Therapy on Platelet Recovery in Pediatric Immune Thrombocytopenic Purpura-Case Series and a Systematic Review

**DOI:** 10.3390/microorganisms8101457

**Published:** 2020-09-23

**Authors:** Tamaki Ikuse, Masanori Toda, Kosuke Kashiwagi, Kimiko Maruyama, Masumi Nagata, Kaori Tokushima, Natsuki Ito, Kazuhide Tokita, Reiko Kyodo, Kenji Hosoi, Keisuke Jimbo, Takahiro Kudo, Toshiaki Shimizu

**Affiliations:** Department of Pediatrics, Juntendo University Faculty of Medicine, 3-1-3 Hongo, Bunkyo-ku, Tokyo 113-8431, Japan; m.toda.gi@juntendo.ac.jp (M.T.); k-kashiwagi@juntendo.ac.jp (K.K.); km-maruyama@juntendo.ac.jp (K.M.); ma-nagata@juntendo.ac.jp (M.N.); k-tokushima@juntendo.ac.jp (K.T.); na-ito@juntendo.ac.jp (N.I.); ka-tokita@juntendo.ac.jp (K.T.); rkyoudo@juntendo.ac.jp (R.K.); khosoi@juntendo.ac.jp (K.H.); kjinbo@juntendo.ac.jp (K.J.); t-kudo@juntendo.ac.jp (T.K.); tshimizu@juntendo.ac.jp (T.S.)

**Keywords:** *Helicobacter pylori*, immune thrombocytopenic purpura, thrombocytopenia, eradication therapy, pediatrics, child

## Abstract

Evidence relating to the efficacy of *Helicobacter pylori* eradication therapy for chronic immune thrombocytopenic purpura (cITP) in childhood is inadequate. The aim of this retrospective study was to determine the efficacy of *H. pylori* eradication therapy for platelet response in pediatric patients with cITP in our hospital, and to perform a systematic review of previous reports about pediatric patients with cITP who were positive for *H. pylori* infection and were treated with *H. pylori* eradication therapy. Analysis of the data of pediatric patients with cITP in our hospital and a systematic review of digital literature databases of studies in pediatric patients with cITP were performed. Data of 33 pediatric patients with cITP from our hospital records showed that the prevalence of *H. pylori* infection and the rate of response to platelet therapy were 15% and 33.3%, respectively. Data of 706 pediatric patients from 18 previous reports showed that the prevalence of *H. pylori* infection and rate of platelet response were 23% and 43.8%, respectively. Eradication therapy for *H. pylori* infection in pediatric cITP patients can be expected to result in a response equivalent to that in the adult population, with fewer adverse effects than other treatments for cITP.

## 1. Introduction

*Helicobacter pylori* (*H. pylori*) is a microaerophilic Gram-negative spiral bacterial pathogen that resides in the stomach. Urease produced by the bacteria protects them from the highly acidic environment. *H. pylori* infection is generally acquired in childhood and typically persists for life. Chronic infection with *H. pylori* is a well-known cause of gastric diseases including chronic active gastritis, atrophic gastritis, peptic ulcer diseases, mucosa associated lymphoid tissue lymphoma, and gastric cancer [1]. Many studies have shown conflicting results relating to the association between *H. pylori* infection and extraintestinal diseases including nutritional deficiencies, such as vitamin B12 deficiency, and hematological pathologies such as iron deficiency anemia and idiopathic or immune thrombocytopenic purpura (ITP) [2,3].

ITP, also known as immune thrombocytopenia, is an immune-mediated acquired disease characterized by thrombocytopenia (peripheral blood platelet count <100,000/µL with normal white blood cell count and hemoglobin), in the absence of other causes or disorders that may be associated with thrombocytopenia [4]. Persistent thrombocytopenia for more than 6 months was previously defined as chronic ITP (cITP) [5]; but recently the definition has been changed to more than 12 months, and cases of thrombocytopenia which are less than 3 months, or between 3 and 12 months from the time of diagnosis are categorized as newly diagnosed ITP and persistent ITP, respectively [4].

Most adults with ITP require one or more therapies and develop cITP [6]. However, most pediatric patients with ITP recover within 3–6 months of presentation, with or without treatment [7,8], and cITP is observed in up to 20% of newly diagnosed ITP cases in children [9,10]. Although a serious bleeding episode including intracranial hemorrhage is known to occur in less than 10% of children with cITP [8], they may require frequent laboratory monitoring in hospital, restriction of physical activities, pharmacologic therapies including prolonged use of glucocorticoids, and surgical interventions including splenectomy.

Since the first report in 1998 [11], there have been numerous reports especially from Japan, Italy, and Spain about the efficacy of treatment for eradication of *H. pylori* in patients with ITP who are positive for *H. pylori* infection [11,12,13,14,15,16,17,18,19,20,21,22]. In Japan, platelet counts have been reported to increase in 40–60% of adult patients with ITP, following *H. pylori* eradication. A meta-analysis also showed a significant increase in platelets after treatment for eradication of *H. pylori* [23], and the increased count was reportedly maintained over the long term [16,17,19,21,24]. *H. pylori*-negative patients did not show an increase in platelet count after *H. pylori* eradication therapy, and *H. pylori*-positive cases may not achieve an increase in platelets or a continuing response in case of unsuccessful eradication therapy [25]. Considering the adverse effects of prolonged corticosteroid use, the benefits and duration of response with high-dose immunoglobulin therapy, the costs of these treatments, and the risk of bleeding and postoperative infection after splenectomy, eradication of *H. pylori* has been the first-line therapy in Japan for adult patients with ITP who are confirmed *H. pylori* positive [26].

Recently, the Japanese Society of Pediatric Gastroenterology, Hepatology, and Nutrition (JASPGHAN) guidelines for the Management of *Helicobacter pylori* Infection in Childhood have recommended eradication therapy for *H. pylori*-infected children with cITP as first-line therapy [27]. However, the 2019 Treatment Guide for Refractory Immune Thrombocytopenia in Children by the Platelet Committee of Japanese Society of Pediatric Hepatology and Oncology does not refer to *H. pylori* infection [28]. These differences in opinion are due to the lack of evidence in the pediatric population for the benefits observed in adult patients. The aim of this study was to perform a retrospective analysis of pediatric patients with cITP in our hospital and a systematic review of previous reports about pediatric cITP published in English and Japanese, to determine the relationship between cITP and *H. pylori* infection in children and assess the efficacy of *H. pylori* eradication therapy in achieving a platelet response in children, especially in Japan.

## 2. Materials and Methods

### 2.1. Patients

Medical records of all patients with the term “thrombocytopenic” or “thrombocytopenia” in their diagnosis at the Juntendo University Hospital between January 2002 and December 2018 were reviewed retrospectively. The patients selected for the study met the following inclusion criteria: (1) age <18 years at the time of diagnosis of ITP and (2) diagnosis of ITP according to the American Society of Hematology criteria [5] based on an initial platelet count <100,000/μL. The exclusion criteria were as follows: (1) thrombocytopenia related to autoimmune disorders, drugs, a family history consistent with inherited thrombocytopenia, human immunodeficiency virus (HIV) infection, hepatitis, congenital heart disease, or pseudothrombocytopenia and (2) previous history of *H. pylori* eradication before the diagnosis of ITP. In this study, we defined cITP as persistent thrombocytopenia for longer than 6 months. Among cITP cases, data of patients who underwent diagnostic tests for *H. pylori* infection were extracted and the results of eradication therapy were reviewed. All subjects gave their informed consent for inclusion before they participated in the study. The study was conducted in accordance with the Declaration of Helsinki, and the protocol was approved by the Institutional Ethics Committee of Juntendo University Hospital (the project identification code: no. 20-184, date of approval: 25 August 2020).

### 2.2. Diagnosis of *H. Pylori* Infection, and Eradication Therapy

*H. pylori* infection was diagnosed using the 13C-urea breath test (UBT), *H. pylori* stool antigen test (HpSA), or serum anti-*H. pylori* IgG antibody test. Based on previously published reports, pediatric patients were considered positive for UBT if a delta over baseline ≥3.5‰ was achieved [29]. The gastric biopsy samples for bacterial culture were obtained endoscopically from patients who tested positive for any of the diagnostic tests for *H. pylori*. Antibiotic sensitivity of *H. pylori* isolates to clarithromycin (CAM), amoxicillin (AMPC), and metronidazole (MNZ) were examined using the dilution method with Mueller–Hinton agar plates containing 5% horse blood. The minimal inhibitory concentration (MIC) breakpoints were defined as follows; ≥1 μg/mL for CAM, >0.125 μg/mL for AMPC, and >8 μg/mL for MNZ [30].

Patients infected with CAM-sensitive strains were prescribed the CAM-based regimen for 7–14 days, which consisted of a proton pump inhibitor (PPI), AMPC (50 mg/kg per day), and CAM (20 mg/kg per day). Patients infected with CAM-resistant strains were prescribed the MNZ-based regimen for 7–14 days, which consisted of PPI, AMPC (50 mg/kg per day), and MNZ (20 mg/kg per day). *H. pylori* eradication was confirmed by UBT or *H. pylori* stool antigen test performed at least 8 weeks after treatment.

### 2.3. Assessment of Treatment Efficacy

We evaluated the treatment efficacy as follows. A response was considered complete if the platelet count increased to at least 100,000/μL within 2 months of initiation of anti-*H. pylori* treatment. Partial response was defined as an increase in platelets to a minimum of 50,000/μL within 2 months of initiation of treatment regardless of whether the patient was on maintenance therapy, or an increased platelet count to more than twice that during the pretreatment period. No response was defined as a platelet count that remained below 50,000/μL or showed no increase.

### 2.4. Systematic Review

#### 2.4.1. Defining the Clinical Question

To identify the association between *H. pylori* infection and cITP in children, we stated the clinical question as follows: can *H. pylori* eradication therapy achieve an increasing platelet response in pediatric cITP patients with *H. pylori* infection?

#### 2.4.2. Identification of Evidence

Electronic databases including PubMed and ICHUSHI (Japan’s largest medical-literature database) were searched in September 2019 using specific keywords. Articles published in English and Japanese were included. For PubMed, we performed a detailed search using (((((“Thrombocytopenia”[Mesh]) OR (“Purpura, Thrombocytopenic, Idiopathic”[Mesh] OR “Purpura, Thrombotic Thrombocytopenic”[Mesh] OR “Purpura, Thrombocytopenic”[Mesh])) OR thrombocytopen [TIAB]) OR ITP [TIAB])) AND (((“Helicobacter pylori”[Mesh]) OR “Helicobacter Infections”[Mesh]) OR pylori[TIAB]). A total of 401 articles were retrieved. For ICHUSHI, we performed a detailed search using (((thrombocytopenia (in Japanese)/TH or thrombocytopenia (in Japanese)/AL) or (thrombocytopenic purpura (in Japanese)/TH or thrombocytopenic purpura (in Japanese)/AL) or (ITP/TA)) and ((Helicobacter/TH or Helicobacter/AL) and pylori/AL) or ([Helicobacter pylori]/TH) or ([Helicobacter infection (in Japanese)]/TH) or (”Helicobacter pylori”/TH and Helicobacter infection (in Japanese)/TH) or (Pylori (in Japanese)/TA) or pylori/TA))) and (PT = except record of proceedings (in Japanese)), and found 527 articles. A total of 928 articles were retrieved from the combined searches. Study references were collected in Endnote x9 (Clarivate Analytics Japan, Minato-ku, Tokyo, Japan).

#### 2.4.3. Study Selection

Two reviewers (TI and TK) independently performed primary screening of the articles based on their titles and abstracts blinded to each other’s decisions. Studies were considered eligible for inclusion if they related to the effects of *H. pylori* eradication on the platelet count in pediatric patients with cITP in randomized-controlled, case-controlled, or retrospective studies. The exclusion criteria were as follows: (1) papers not written in English or Japanese, (2) research not involving humans (e.g., in vitro or animal research), and (3) research including patients with secondary thrombocytopenia due to autoimmune disorders, drugs, family history consistent with inherited thrombocytopenia, HIV infection, hepatitis, or congenital heart disease. Selection or rejection of articles that could not be decided through primary screening were retained for secondary screening. The results of the two reviewers were crosschecked, and a dataset for secondary screening was prepared which included 153 full-text articles. Two reviewers (TI and TK) independently read the full-text articles, and performed the secondary screening. Articles that included evaluation of the effects of *H. pylori* eradication on the platelet count in pediatric patients with cITP were selected independently, and the results of the two reviewers were crosschecked. Where the two reviewers had different opinions that could not be resolved by discussion, a third person was invited to make a final decision. All decisions were recorded in Microsoft Excel (Microsoft, Redmond, WA, USA).

#### 2.4.4. Data Extraction

Two reviewers (TI and TK) independently extracted data from each study using a data extraction form that was designed in advance. The following information was extracted: first author, year of publication, study design, definition of cITP and platelet response, age of patients, diagnostic tests of *H. pylori* infection, eradication regimens, duration of eradication therapy, confirmatory test of eradication, and confirmation period of platelet response. Where the two reviewers had different opinions that could not be resolved by discussion, a third person was invited to make a final decision. All decisions were recorded in Microsoft Excel (Microsoft, Redmond, WA, USA).

#### 2.4.5. Study Quality Assessment

Methodological quality of included RCTs was assessed using Jadad score [31] the following items: (1) randomization; (2) blinding; (3) withdrawals and dropouts. Nonrandomized studies (NRCTs) including cohort or case control study were assessed in accordance with the Newcastle-Ottawa scale (NOS) [32]. They were assessed by selection, comparability, and exposure or outcome.

## 3. Results

### 3.1. Retrospective Analysis

We evaluated 642 pediatric patients in whom the term “thrombocytopenic” or “thrombocytopenia” was used in the diagnosis. After exclusion of thrombocytopenia due to other causes, 80 patients with ITP between January 2002 and December 2018 were selected. Thirty-three patients showed persistent thrombocytopenia for more than 6 months, meeting the criteria for cITP (Table 1).

Diagnostic evaluations for *H. pylori* were performed in 20 patients, and three of them had at least one positive diagnostic test result. The results of *H. pylori* culture in all three patients were positive; thus, the prevalence of *H. pylori* infection among 20 pediatric patients with cITP was at least 15% even in the unlikely event that false-negative cases were included.

All three patients received *H. pylori* eradication therapy, which was successful in all of them after the first course of antimicrobial treatment. One patient showed a complete response and continues to have a satisfactory platelet count more than 12 months following eradication therapy. No significant increase in the platelet count was seen in the other two patients. Therefore, the response rate in our patients was 33.3%.

### 3.2. Remission Case Report

A 10-year-old girl diagnosed with cITP was admitted with recurrent purpura, nasal bleeding, and thrombocytopenia for more than a year. She also complained of recurrent episodes of epigastric pain about twice a week, for several years. Investigations performed previously at another hospital showed persistent low platelet count below 50,000/μL, elevated platelet associated immunoglobulin G (196.0 ng/10^7^ cells), and normal results of white and red blood cell counts and bone marrow examination (nucleated cells: 18.9 × 10^10^/L, megakaryocytes: 150 × 10^6^/L, M/E ratio: 1.46). She had been prescribed several courses of prednisolone, intravenous immunoglobulin, and methylprednisolone when the platelet counts fell below 20,000/μL and was symptomatic. However, no significant response was achieved with these treatments.

At our hospital, after complaining of chronic epigastric pain and giving a positive family history of *H. pylori* infection, she was investigated for the presence of *H. pylori* infection. UBT and serum anti-*H. pylori* IgG antibody tests were positive. Esophagogastroduodenoscopy revealed nodular gastritis on the antral mucosa, and *H. pylori* was detected on bacterial culture of the biopsy sample. After confirmation of antibiotic sensitivity to AMPC and CAM, eradication therapy comprising AMPC (1500 mg/day), CAM (400 mg/day), and lansoprazole (45 mg/day) was prescribed for 14 days. The patient’s platelet count increased dramatically and reached 187,000/μL 4 weeks after *H. pylori* eradication. She had received no other treatment for cITP for a month before starting *H. pylori* eradication therapy. She maintained complete remission over a year and was advised no further follow-ups.

### 3.3. Systematic Review

#### 3.3.1. Study Selection and Characteristics of Included Studies

**Figure 1:**
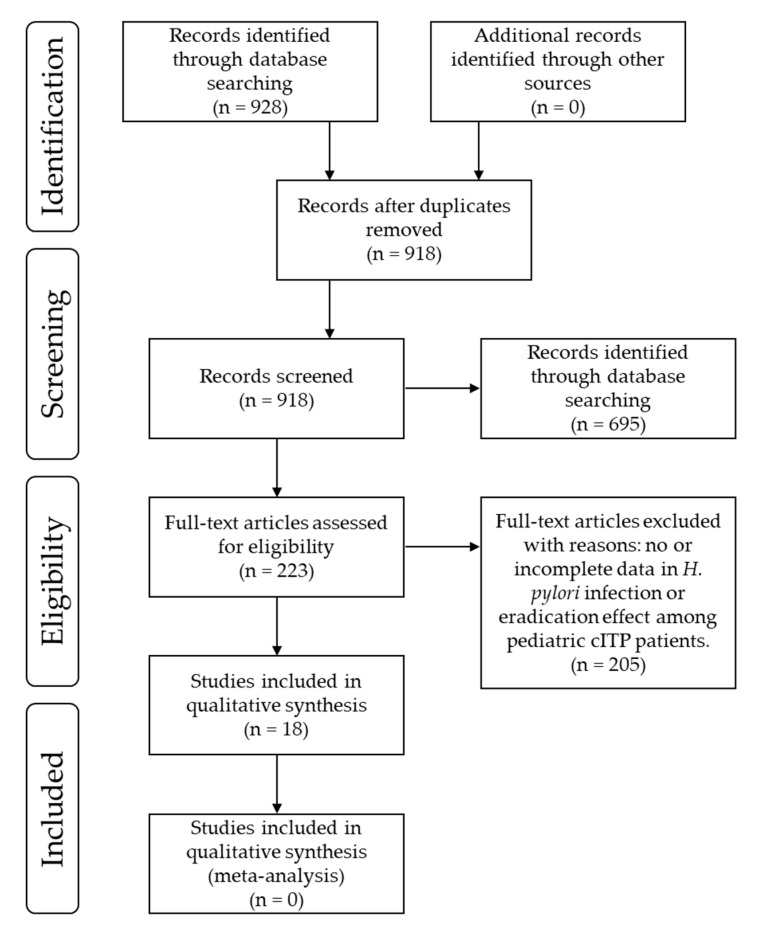
Flow of study identification, inclusion, and exclusion.

A flow diagram of study identification, inclusion, and exclusion is provided in Figure 1. The searches of PubMed and ICHUSHI using the defined terms yielded a total of 928 records. After 10 duplicates were removed, 918 records remained for the title and abstract screen. A total of 695 records were excluded based article type (reviews, case reports, irrelevant experiments, incomplete data). After full-text assessment of the 223 remaining records, we finally identified and reviewed 18 studies [33,34,35,36,37,38,39,40,41,42,43,44,45,46,47,48,49,50] consisting of two RCTs and 16 NRCTs (case-controlled, or retrospective studies, or case series) with sufficient data relating to *H. pylori* infection and the efficacy of *H. pylori* eradication therapy in pediatric patients with cITP. Characteristics of the 18 studies are summarized in Table 2. They were published between 2003 and 2015. We extracted the data of 706 pediatric patients with cITP persistent thrombocytopenia (platelet count <150,000/μL) for longer than 6 months, including 164 patients with *H. pylori* infection from these previous studies (Table 2).

#### 3.3.2. Risk of Bias

The two RCTs demonstrated an acceptable level of quality according to Jadad score, 3 or higher. The Newcastle-Ottawa Scale scores of the six cohort and two case control studies ranged from 5 to 9, indicating moderate risks of bias.

#### 3.3.3. The Prevalence of *H. Pylori* Infection and Platelet Response Rate

The total prevalence of *H. pylori* infection among all 726 cITP patients including our patients was 23.0%, and a wide variability in prevalence from 0% to 43.5% depending on the reports was observed. Among 167 cITP patients with *H. pylori* infection, 132 patients were prescribed eradication therapy. The platelet response rates after eradication therapy also showed a wide variability from 0% to 100%, and the total response rate was 43.8%; a significant increase in platelet count following *H. pylori* eradication therapy was observed in 60 patients. When limited to the cases in Japan, the prevalence of *H. pylori* infection was 11.6%; 11 of 95 cITP patients tested positive for *H. pylori*, and the platelet response rate was 42.9%; however, it should be noted that the data were based on only three responders among seven cITP patients.

## 4. Discussion

ITP is an autoimmune disease that causes thrombocytopenia due to the production of autoantibodies against platelets, and is often triggered by some infection or vaccination in childhood. Although 80–90% of pediatric patients with ITP recover spontaneously, some cases are refractory to the administration of glucocorticoid and immunoglobulin and may experience severe bleeding. There are regional, ethnic, and racial differences; however, there is much evidence in adults for an association between *H. pylori* infection and ITP. The mechanism by which *H. pylori* infection causes ITP has been investigated from various perspectives. Takahashi et al. reported that cross-reaction of the platelet membrane glycoprotein (GP) IIb/IIIa, GP Ib/IX, GP Ia/IIa, and GP VI, the antigen corresponding to antiplatelet autoantibodies, with the *H. pylori* constituent protein CagA caused thrombocytopenia [22]. Asahi et al. showed that *H. pylori* infection resulted in inhibition of the Fcγ receptor on peripheral blood monocytes, and led to increased production of antiplatelet antibodies, thereby accelerating platelet turnover through decreased expression of FcγRIIB [12]. However, the pathogenesis of ITP and its causal relationship with *H. pylori* infection is still not clear.

Given that children have a lower prevalence of *H. pylori* infection and lower incidence of refractory ITP than adults, data from previous reports are limited to a small number of patients. Although Russo et al. [35] in Italy, Treepongkaruna et al. [38] in Thailand, and Brito et al. [34] in Brazil reported the results of prospective, multicenter, and interventional trials investigating the relationship between *H. pylori* infection and ITP in childhood, it is still difficult to accumulate comprehensive evidence with an adequate sample size to determine the efficacy of *H. pylori* eradication therapy in children with cITP who test positive for *H. pylori*.

Thus, these suggestions are based on extremely limited information, although the prevalence of *H. pylori* infection among children with cITP may be slightly higher than that in the general pediatric population as observed in Italy, Taiwan, and Japan. The prevalence reported in systematic reviews from Japan (11.6%) was about the same as, or slightly higher than, the prevalence in other children of the same age in Japan, in whom it was approximately 5–10%. This trend was also observed in Japanese adults, among whom the prevalence of *H. pylori* infection in cITP patients (69%) was slightly higher than that in the general population of the same age group (about 60%) [20]. This lack of a significant difference in the infection rate indicates that ITP is a multifactorial disease and that *H. pylori* infection is just one of the causes of ITP in adults and children.

A wide variability in the rates of platelet increase from 0% to 100% after *H. pylori* eradication therapy was observed in the review of previous reports. However, when all previous cases were combined, the mean rate of platelet increase was 43.8%. This result proves the existence of a subset of pediatric cITP patients who would benefit from *H. pylori* eradication therapy, and suggests that a response rate equivalent to that observed in the adult population (40–60%) can be expected [20]. Therefore, *H. pylori* eradication therapy should be considered as the first treatment for cITP in children with *H. pylori* infection, given the adverse effects of systemic glucocorticoid administration and splenectomy. The cost of treatment and fewer side effects with *H. pylori* eradication therapy makes it a favorable first choice.

Our study has several inherent limitations that need to be discussed. First, this systematic review included a limited number of studies with a small sample size indicating moderate risks of bias, and there were only three prospective, multicenter, interventional trials [34,35,38]. Second, the indication for *H. pylori* diagnostic testing in cITP pediatric patients depended on the decision of the main attending physician in our retrospective analysis. Thus, 13 of 33 pediatric cITP patients in our hospital were not evaluated for *H. pylori* infection. Further multicenter studies or large randomized controlled trials are necessary to establish an adequate evidence base for this therapy in pediatric patients with cITP.

## 5. Conclusions

This study, based on a systematic review of pediatric cITP reports from Japan and abroad, and a retrospective analysis of 33 cITP patients from our hospital, shows that the efficacy of *H. pylori* eradication therapy in inducing a platelet response among pediatric patients with cITP is equivalent to that seen in adults. The study suggests that *H. pylori* eradication therapy should be considered as the first-line treatment for cITP in children with *H. pylori* infection.

## Figures and Tables

**Table 1:** Characteristics of 33 pediatric patients with chronic immune thrombocytopenic purpura (cITP).

	Number of Patients	Mean Age, Standard Deviation, and Range (Years)
Total	33	9.05 ± 4.35 (0.67–15.92)
Gender		
Male	15	9.61 ± 3.97 (0.67–14.67)
Female	18	8.57 ± 4.70 (2.33–15.92)
*H. pylori* infection		
Evaluated	20	8.34 ± 4.28 (0.67–15.91)
Positive	3	9.83 ± 3.25 (6.5–13.0)
Negative	17	8.08 ± 4.48 (0.67–15.92)
Unknown	13	10.13 ± 4.37 (3.25–15.67)
Eradication therapy	Success rate: 100%	
Responder	1	10.0 ^†^
Nonresponder	2	6.5 ^†^, 13.0 ^†^

^†^: individual values.

**Table 2:** Summary of the 18 studies included and pediatric patients with cITP in our hospital.

First Author (Year)	Country	Prevalence of *H. pylori* Infection		Response to *H. pylori* Eradication Therapy ^†^		Study Design (Risk of Bias Score)	Definition of cITP		Platelet Count Defined As Platelet Response (×10^4^/μL)	Follow-Up Duration (Months)
							Platelet Count (×10^4^/μL)	Duration (Months)		
Amiri M (2015) [33]	Iran	43.5%	(10/23 cases)	75.0%	(6/8 cases)	CO (NOS: 9)	<10	>12	Compared statistically	6
Brito HS (2015) [34]	Brazil	25.9%	(22/85 cases)	61.5%	(8/13 cases)	RCT (Jadad: 3)	<15	>6	CR > 15 PR > 5 and Δ > 2	12
Russo G (2011) [35]	Italy	25.8%	(50/194 cases)	39.4%	(13/33 cases)	CO (NOS: 8)	<10	>12	CR > 15 PR > 5 and Δ > 3	12
Ferrara M (2009) [36]	Italy	33.3%	(8/24 cases)	100%	(8/8 cases)	CO (NOS: 8)	<50	>6	CR > 15 PR > Δ > 5	12
Maghbool M (2009) [37]	Iran	16.7%	(5/30 cases)	100%	(5/5 cases)	CCS (NOS: 8)	<10	>6	CR > 15 PR > 5	12
Treepongkaruna S (2009) [38]	Thailand	29.1%	(16/55 cases)	14.3%	(2/14 cases)	RCT (Jadad: 3)	<10	>6	>10	12
Bisogno G (2008) [39]	Italy	33.3%	(8/24 cases)	37.5%	(3/8 cases)	CO (NOS: 9)	<10	>6	CR > 15 PR > 5 and Δ > 3	>6
Hamidieh AA (2008) [40]	Iran	12.9%	(4/31 cases)	0%	(0/4 cases)	CS (N/A)	<15	>6	CR > 15 PR > Δ > 5	>6
Miyajima Y (2008) [41]	Japan	7.1%	(1/14 cases)	ND		CSS (N/A)	<10	>6	>15	3
Kato F (2007) [42]	Japan	27.3%	(3/11 cases)	33.3%	(1/3 cases)	CS (N/A)	<10	>6	>15	12
Loffredo G (2007) [43]	Italy	20.5%	(8/39 cases)	0%	(0/7 cases)	CO (NOS: 6)	<10	>6	>12	12
Neefjes VM (2007) [44]	The Netherlands	6.4%	(3/47 cases)	100%	(3/3 cases)	CCS (NOS: 5)	<10	>12	CR > 15 PR > 5 and twice	>6
Wu KS (2007) [45]	Taiwan	40.0%	(2/5 cases)	ND		CSS (N/A)	<50	>6	N/A	N/A
Hayashi H (2005) [46]	Japan	20.0%	(2/10 cases)	100%	(1/1 cases)	CS (N/A)	<10	>6	>15	12
Yetgin S (2005) [47]	Turkey	31.4%	(11/35 cases)	0%	(0/9 cases)	CS (N/A)	<50	>6	>5 within 12 months	12
Jaing TH (2003) [48]	Taiwan	40.9%	(9/22 cases)	55.6%	(5/9 cases)	CO (NOS: 7)	<50	>6	CR > 15 PR > 5	16
Rajantie J (2003) [49]	Finland	0%	(0/17 cases)	ND		CS (N/A)	N/D	N/D	N/A	N/A
Sakai M (2003) [50]	Japan	5.0%	(2/40 cases)	ND		CSS (N/A)	<10	>6	N/A	N/A
This study	Japan	15%	(3/20 cases)	3.3%	(1/3 cases)	CS (N/A)	<10	>6	CR > 15, PR > 5 or twice within 2 months	12
Total ^†^		23.0%	(167/726 cases)	43.8%	(56/128 cases)					

CCS: case control study, cITP: chronic immune thrombocytopenia, CO: cohort study, CR: complete response, CS: case series, CSS: cross sectional study, *H. pylori*: *Helicobacter pylori*, Jadad: Jadad score [31], N/A: not applicable, N/D: not described, NOS: Newcastle-Ottawa Scale [32], PR: partial response, RCT: randomized control trial. Δ: describing the platelet increase. Response includes both complete response and partial response. ^†^: result of a pooled data analysis.
